# Editorial: Insights in sleep-related movement disorders and parasomnias

**DOI:** 10.3389/frsle.2024.1461464

**Published:** 2024-08-08

**Authors:** Ambra Stefani, John Winkelman

**Affiliations:** ^1^Medical University Innsbruck, Innsbruck, Austria; ^2^Harvard Medical School, Boston, MA, United States

**Keywords:** neuroscience, sleep, sleep-related movement disorders, restless legs syndrome, RLS, REM sleep behavior disorder, NREM parasomnia, RBD

This Research Topic focuses on novel insights into sleep-related movement disorders and parasomnias, highlighting current challenges and future perspectives in the field. The field is constantly evolving, with advances in our understanding of restless legs syndrome (RLS) pathophysiology and changes in treatment recommendations, novel insights into sleep-related movement disorders and their clinical relevance, including the description and neurophysiological characterization of new sleep-related movement disorders, as well as a rapidly changing scenario in parasomnias, in particular for REM sleep behavior disorder (RBD), but also the presence and temporal dynamics of sleep changes in synucleinopathies.

This Research Topic includes four articles, two original research articles and two reviews.

One original work filled a gap in the field of RLS, assessing the prevalence of the disease in Saudi Arabia through a nationwide survey. RLS was commonly reported, with a 11.9% prevalence, and was associated with anxiety and depression, while female gender and depression were associated with RLS causing significant daytime impairment. One additional review focused on sleep-related movement disorders in older adults, addressing specific considerations on diagnosis and management in this populations, taking into account comorbidities and polypharmacy.

Two articles focused on patients with Parkinson's disease (PD). One original article cross-sectionally evaluated sleep quantitative EEG in patients with PD with and without RBD, relating these findings to cognitive performance. PD patients with RBD showed worse cognitive performance and had alteration in sleep quantitative EEG, compared to PD patients without RBD. Worse cognitive score was predicted by lower sleep spindle density. A narrative review provided an update on excessive daytime sleepiness (EDS) in PD, with insights on how to disentangle EDS secondary to medications or other sleep disorders from primary EDS, which is a key feature of the Park-sleep subtype of PD. The authors propose a multistep approach to evaluate EDS in PD and provide specific considerations for EDS management in PD.

Taken together, this Research Topic provides novel insights into the field of sleep-related movement disorders and parasomnias, highlighting current challenges and proposals to address them in the future.

## Author contributions

AS: Writing – original draft, Writing – review & editing. JW: Writing – original draft, Writing – review & editing.

